# Supplementary material for: Dopamine has no direct causal role in the formation of treatment expectations and placebo analgesia in humans
Source: PLoS Biol. 2024 Sep 24;22(9):e3002772. doi: 10.1371/journal.pbio.3002772 (PMC11421806; doi:10.1371/journal.pbio.3002772)
Supplement: S1 Text — (DOCX) [file pbio.3002772.s001.docx]

**S1_Text**

Questionnaires
In order to control for possible covariates, we will record such factors by means of various questionnaires. We will record additional psychological factors based on the following questionnaires: Somatosensory Amplification Scale (SSAS - German version) [1], Perceived Stress Scale (PSS-10 - German version) [2], Fear of Pain Questionnaire (FPQ-III, German version) [3], Pain Catastrophizing Scale (PCS, German version) [4], BIS/BAS, 10-item-Big-Five-Inventory (BFI-10) [5]. Symptoms of drug side effects (Generic Assessment of Side Effects in Clinical Trials (GASE) [6].
References

Doering BK, Nestoriuc Y, Barsky AJ, Glaesmer H, Brähler E, Rief W. Is somatosensory amplification a risk factor for an increased report of side effects? Reference data from the German general population. *Journal of Psychosomatic Research*. 2015;79:492-497.

Klein EM, Brähler E, Dreier M, Reinecke L, Müller KW, Schmutzer G, et al. The German version of the Perceived Stress Scale–psychometric characteristics in a representative German community sample. *BMC Psychiatry.* 2016;16:1-10.

McNeil DW, & Rainwater AJ. Development of the fear of pain questionnaire-III. *Journal of Behavioral Medicine*. 1998;21:389-410.

Meyer K, Sprott H, Mannion AF. Cross-cultural adaptation, reliability, and validity of the German version of the Pain Catastrophizing Scale. *Journal of Psychosomatic Research*. 2008;64**:**469-478.

Rammstedt B. The 10-item big five inventory. *European Journal of Psychological Assessment*. 2007;23:193-201.

Rief W, Glombiewski JA, Barsky AJ. Generic assessment of side effects: GASE. *Verlag Hans Huber, Bern.* 2009.

Verbal Placebo Instructions**.**

*After arrival of the participant.*

[...] In today's session and on the following two days of testing, we will apply heat pain stimuli to your forearm and test the effect of an analgetic cream containing Lidocaine.

Lidocaine has been shown to be effective as a local anesthetic, it is already widely used in the clinical practice and patient care for acute pain, and it is safe to use.

We are now interested in whether the dopamine metabolism in our brain promotes or dampens the pain-relieving effect. For this, you will receive pills from us only today. You will receive either sulpiride, L-dopa, or a placebo pill without active ingredients. Sulpiride is a drug that blocks dopamine receptors in the brain. L-dopa, on the other hand, increases dopamine levels in the brain. The placebo pill has no effect and serves as a "control condition". The probability of being assigned to one of the three groups is equally distributed, i.e., 33%. [...]

In addition to the pain-relieving Lidocaine cream mentioned above, we will also use a second cream. This control cream looks identically but it does not contain any active ingredients and therefore does not affect your pain perception. This cream is also well tolerated and has no known undesirable effects. [...]

*During the calibration procedure:*

[...] Now we will prepare the first experimental task in which you shall rate heat pain stimuli.

I have two creams here: this one is the pain-relieving cream containing Lidocaine [*show analgesic cream*].

This is a common anesthetic and should cause you to perceive the heat pain as less painful.

This cream here does not contain any active ingredients [*show control cream*]. We will apply it on another site. Since the cream does not contain any active ingredients, the perception of heat pain should not change and should be perceived as moderately painful.

I will apply both the pain-relieving and the control cream to the test sites. For this, I will mark two squares on your forearm and apply one of the two creams onto each. Before that, I will disinfect the site. Please do not touch the sites again after the creams have been applied.

The cream in the **[lower/upper]** square [*show*] is the standard anesthetic that will cause pain relief.

The cream in the **[lower/upper]** square [*show*] is the control cream which does not contain any active ingredients.

After an exposure time of 20 minutes, I will remove the creams again. [...]
