# Supplementary material for: Dopamine has no direct causal role in the formation of treatment expectations and placebo analgesia in humans
Source: PLoS Biol. 2024 Sep 24;22(9):e3002772. doi: 10.1371/journal.pbio.3002772 (PMC11421806; doi:10.1371/journal.pbio.3002772)
Supplement: S1 Table — (A) Model comparison. Note that models include subject and random slopes for all repeated measures factors. (B) Analysis of effects showing the inclusion Bayes factors (BFincl) of the model terms. (C) Summary of model averaged posteriors showing estimated marginal means, standard deviations, and 95% credible intervals for all factor levels. (DOCX) [file pbio.3002772.s002.docx]

| 1. **Model Comparison** | | | | | |
| --- | --- | --- | --- | --- | --- |
| **Models** | **P(M)** | **P(M\|data)** | **BF_M_** | **BF_10_** | **error %** |
| Null model (incl. subject and random slopes) | 0.200 | 0.111 | 0.499 | 1.000 |  |
| experimental condition | 0.200 | 0.687 | 8.780 | 6.199 | 0.876 |
| experimental condition + group | 0.200 | 0.165 | 0.791 | 1.490 | 3.941 |
| medication | 0.200 | 0.023 | 0.094 | 0.207 | 3.343 |
| experimental condition + medication + experimental condition ✻  medication | 0.200 | 0.014 | 0.057 | 0.128 | 6.816 |
|  |  |  |  |  |  |

S1_Table. **Detailed results of Bayesian quantification of evidence for Hypothesis 1 from JASP output.**

| 1. **Analysis of Effects** | | | | | |
| --- | --- | --- | --- | --- | --- |
| **Effects** | **P(incl)** | **P(excl)** | **P(incl\|data)** | **P(excl\|data)** | **BF_incl_** |
| experimental condition | 0.600 | 0.400 | 0.866 | 0.134 | 4.318 |
| medication | 0.600 | 0.400 | 0.202 | 0.798 | 0.169 |
| experimental condition ✻  medication | 0.200 | 0.800 | 0.014 | 0.986 | 0.057 |

| 1. **Model Averaged Posterior Summary** | | | | | |
| --- | --- | --- | --- | --- | --- |
|  | | | | **95% Credible Interval** | |
| **Variable** | **Level** | **Mean** | **SD** | **Lower** | **Upper** |
| Intercept |  | 50.672 | 1.241 | 48.168 | 53.139 |
| experimental condition | Control | 1.504 | 0.537 | 0.415 | 2.557 |
|  | Placebo | -1.504 | 0.537 | -2.642 | -0.556 |
| medication | DOPA | 1.097 | 1.516 | -2.009 | 4.202 |
|  | INA | -1.391 | 1.536 | -4.556 | 1.501 |
|  | SUL | 0.294 | 1.500 | -2.824 | 3.244 |
| experimental condition ✻  medication | Control & DOPA | 0.219 | 0.701 | -1.207 | 1.616 |
|  | Control & INA | 0.311 | 0.711 | -1.105 | 1.757 |
|  | Control & SUL | -0.530 | 0.716 | -1.983 | 0.895 |
|  | Placebo & DOPA | -0.219 | 0.701 | -1.630 | 1.193 |
|  | Placebo & INA | -0.311 | 0.711 | -1.770 | 1.092 |
|  | Placebo & SUL | 0.530 | 0.716 | -0.909 | 1.970 |
