# Supplementary material for: Dopamine has no direct causal role in the formation of treatment expectations and placebo analgesia in humans
Source: PLoS Biol. 2024 Sep 24;22(9):e3002772. doi: 10.1371/journal.pbio.3002772 (PMC11421806; doi:10.1371/journal.pbio.3002772)
Supplement: S3 Table — (A) Model comparison. Note that models include subject and random slopes for all repeated measures factors. (B) Analysis of effects showing the inclusion Bayes factors (BFincl) of the model terms. (C) Summary of model averaged posteriors showing estimated marginal means, standard deviations, and 95% credible intervals for all factor levels. (DOCX) [file pbio.3002772.s004.docx]

**S3_Table. Detailed results of Bayesian quantification of evidence for Hypothesis 3 from JASP output.**

| 1. **Model Comparison** | | | | | |
| --- | --- | --- | --- | --- | --- |
| **Models** | **P(M)** | **P(M\|data)** | **BF_M_** | **BF_10_** | **error %** |
| Null model (incl. subject and random slopes) | 0.200 | 0.001 | 0.004 | 1.000 |  |
| rating timepoint | 0.200 | 0.773 | 13.648 | 738.370 | 0.965 |
| rating timepoint + medication | 0.200 | 0.207 | 1.047 | 198.077 | 1.381 |
| rating timepoint + medication + rating timepoint ✻  medication | 0.200 | 0.018 | 0.073 | 17.080 | 2.588 |
| medication | 0.200 | 2.522×10^-4^ | 0.001 | 0.241 | 0.562 |

| 1. **Analysis of Effects** | | | | | |
| --- | --- | --- | --- | --- | --- |
| **Effects** | **P(incl)** | **P(excl)** | **P(incl\|data)** | **P(excl\|data)** | **BF_incl_** |
| rating timepoint | 0.600 | 0.400 | 0.999 | 0.001 | 512.305 |
| medication | 0.600 | 0.400 | 0.226 | 0.774 | 0.194 |
| rating timepoint ✻  medication | 0.200 | 0.800 | 0.018 | 0.982 | 0.073 |

| 1. **Model Averaged Posterior Summary** | | | | | |
| --- | --- | --- | --- | --- | --- |
|  | | | | **95% Credible Interval** | |
| **Variable** | **Level** | **Mean** | **SD** | **Lower** | **Upper** |
| Intercept |  | 6.158 | 0.127 | 5.894 | 6.399 |
| rating timepoint | preCOND | -0.320 | 0.075 | -0.483 | -0.173 |
|  | preT1 | 0.320 | 0.075 | 0.164 | 0.464 |
| medication | DOPA | 0.001 | 0.161 | -0.332 | 0.314 |
|  | INA | 0.207 | 0.167 | -0.120 | 0.539 |
|  | SUL | -0.208 | 0.166 | -0.554 | 0.101 |
| rating timepoint ✻  medication | preCOND & DOPA | -0.036 | 0.098 | -0.234 | 0.156 |
|  | preCOND & INA | -0.032 | 0.097 | -0.226 | 0.160 |
|  | preCOND & SUL | 0.068 | 0.098 | -0.121 | 0.266 |
|  | preT1 & DOPA | 0.036 | 0.098 | -0.158 | 0.232 |
|  | preT1 & INA | 0.032 | 0.097 | -0.162 | 0.225 |
|  | preT1 & SUL | -0.068 | 0.098 | -0.268 | 0.119 |
