# Supplementary material for: Dopamine has no direct causal role in the formation of treatment expectations and placebo analgesia in humans
Source: PLoS Biol. 2024 Sep 24;22(9):e3002772. doi: 10.1371/journal.pbio.3002772 (PMC11421806; doi:10.1371/journal.pbio.3002772)
Supplement: Attachment — Submitted filename: Response to editor_RR_Stage1_211203.pdf [file pbio.3002772.s008.pdf]

Response to Editor

We thank the Academic Editor for the positive evaluation of our manuscript and the constructive advice. Below, we provide a point-by-point reply. We also explain some adaptions we made to our hypotheses and analysis plan which we believe further improve the quality of the planned study and manuscript. We hope that our manuscript is now suitable to be sent out for peer review and we are looking forward to the reviewers' comments and suggestions.

Point 1)

> *"My main concern is that the analysis plans don't align sufficiently clearly with the hypotheses or power analysis, and I suspect may be overcomplicated, with the consequence that the study may be underpowered to detect differential effects between cells of d=0.3. For example, if I understand correctly, Hypothesis 1 is two independent t-tests (sulpride vs control; L-dopa vs control) but the analysis plan is for a 2 x 3 mixed ANOVA."*

Our main hypothesis (hypothesis 1) indeed assumes a differential effect of medication on the induced placebo effect which could in principle be driven by any of the medication groups or a combination of groups. We are therefore not a priori focussing on specific comparisons (such as the contrast between sulpiride vs. control or L-dopa vs. control).

Any interaction between medication and the induced placebo effect would be novel and of interest for the interpretation of the dopaminergic involvement in the development of placebo analgesia. We therefore believe that an interaction analysis between the factors *medication* (i.e., levels sulpiride, L-dopa and inactive pill) and *experimental condition* (i.e., levels placebo or control) in a 3 x 2 mixed ANOVA would be the correct statistical approach to test our main hypothesis. Importantly, with our planned sample size of N = 165, our study would be rather overpowered than underpowered for this test, as explained in the manuscript (see *Methods* section, *Sampling Plan*):

"Previous studies have reported medium to large effects of pharmacological interventions that acted directly on PA. For instance, pharmacologically blocking opioidergic transmission with naloxone exhibited a large effect in decreasing PA (d = 0.69 [6][1]). With regard to the DA system, the work from ref. [14] [[2]] suggests a medium to large contribution of dopaminergic transmission on the formation of PA, since DA activity in the NAc explained PA well (r = 0.5, R^2 = 25%). Yet, it remains to be investigated whether an active pharmacologic manipulation during the conditioning phase can exert similarly large effects on PA. The sample size for our study is therefore powered to even detect a small effect (Cohen's d *of 0.3) of* medication *on PA."*

In case of a significant interaction (F-test of the ANOVA), post-hoc tests will be used to identify the factor combination(s) driving this interaction effect. Please note that hypotheses 2 and 3 are now considered

“exploratory” to reflect the emphasis on hypothesis 1 to which we have directed the sample size calculation.

Point 2)

> *“(….) and I didn't understand how the within-subjects conditioning variable (placebo vs control) is taken into account in the hypothesis (e.g., through a differential subtraction?)”*

Based on the rationale provided above, both factor levels of the within-subject factor *experimental condition* (e.g., placebo vs. control) will be entered into the ANOVA, as this represents a well-established analysis strategy in the field of placebo research [1, 3-5].

Point 3)

> *“I had similar concerns about the other hypotheses.”*

We have now simplified the statistical model for hypothesis 3 (of the original manuscript) and now consistently use the same ANOVA model as in hypothesis 1, with the difference that *pain ratings* of day 8 are modelled instead of day 2 pain ratings (see Fig. R2 below, and *Introduction* and *Analysis Plan* sections of the revised manuscript). Please also note that hypothesis 2 and 3 of the original manuscript have been swapped to better reflect their logical order (see Fig. R2, R3).

Hypothesis 1 and hypothesis 2 both focus on the investigation of the pharmacologic manipulation of placebo analgesia and will be tested using the same statistical model with the dependent variable (DV) *pain rating* of the two test sessions (see Fig. R1, R2). For hypothesis 1, we will use *pain ratings* of test session 1 (on day 2), whereas for hypothesis 2, we will test pain ratings of test session 2 (on day 8). In both cases, we will fit a mixed 3 x 2 ANOVA with the between-subject factor *medication* (L-dopa vs. sulpiride vs. inactive pill) and the within-subject factor *experimental condition* (placebo vs. control). Please note that we have renamed the *experimental condition* factor and now specify the corresponding levels in all hypotheses to improve clarity.

Hypothesis 3 focuses on the potential influence of the medication on the establishment of treatment expectation towards the placebo cream. To this end, we will fit a mixed 3 x 2 ANOVA with the factors *medication* (L-dopa vs. sulpiride vs. inactive pill) and *rating timepoint* (pre vs. post conditioning) to compare EXPECT ratings (dependent variable) before and after the conditioning session between different medications.

Point 4)

"The authors should make clear, for each hypothesis, the predicted pattern of results in graphical form, and then ensure that the statistical tests and sampling plan map directly on to that predicted pattern specifically."

We thank the Editor for this suggestion. Please find a visualisation of our design and the potential result pattern. If deemed suitable, these could be added to the manuscript.

Figure R1:

(a)

experimental condition „Treatment"	*medication*		
	L-dopa	sulpiride	inactive pill
	n	n	n
placebo „analgesic cream"	55	55	55
control „control cream"	55	55	55

(b)

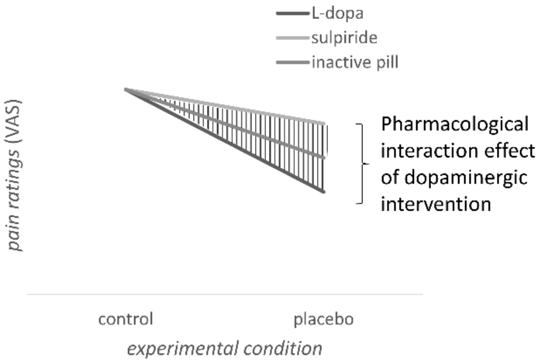

Fig. R1. Hypothesis 1. Group design (a) and potential effects on pain ratings of day 2 for the main hypothesis 1 (b).

Figure R2:

(a)

experimental condition „Treatment"	*medication*		
	L-dopa	sulpiride	inactive pill
	n	n	n
placebo „analgesic cream"	55	55	55
control „control cream"	55	55	55

(b)

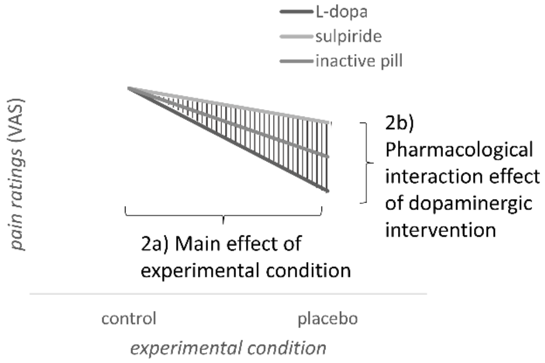

Fig. R2. Hypothesis 2a and 2b. Group design (a) and hypothesized effects on pain ratings of day 8 for hypothesis 2a and 2b (b).

Figure R3:

(a)

rating timepoint	*medication*		
	L-dopa	sulpiride	inactive pill
	n	n	n
pre conditioning (Day 1)	55	55	55
post conditioning (Day 2)	55	55	55

(b)

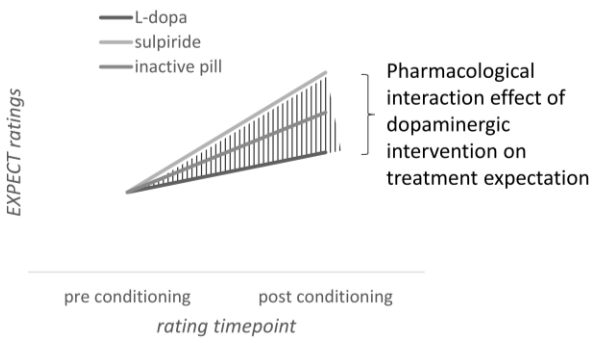

Fig. R3. Hypothesis 3. Group design (a) and hypothesized effects on EXPECT ratings before and after the conditioning session (b).

Point 5)

> *"For instance, the power analysis for a 2 x 3 ANOVA will return the power to detect *any* interaction, when only one of several possible interaction patterns would support the hypothesis. Therefore power is overestimated."*

Please see the response to the Editor's Point 1). As pointed out there, our study is powered to best address our main hypothesis (hypothesis 1) for frequentist statistics. The power of the remaining hypotheses tests (i.e., of hypothesis 2 and 3) is dependent on this predetermined sample size, which has been chosen to be sensitive for small effect sizes of interaction effects in our ANOVA models.

Point 6)

> *"Unless I'm mistaken, I think that all of the authors' hypotheses can be reduced to combinations of pairwise comparisons (e.g. between difference scores), which might be best addressed using Bayesian hypothesis tests (dispensing with p values entirely). But the design isn't sufficiently clear at this stage to say for certain either way."*

We agree that simple pairwise comparisons of difference scores (e.g., differences of pain ratings on the control and placebo site) could be used. However, such approach would not allow for specific conclusions regarding the factor level(s) driving the effect because significant result for such difference scores could in principle be based on differences in ratings for the control site. Given the rationale provided in **point 1),** our comprehensive approach is aimed at detecting *any* interaction, which would be regarded as a novel finding. We therefore believe that a mixed ANOVA that includes all levels of the factors of interest (i.e., levels sulpiride, L-dopa and inactive pill for *medication and* levels placebo

or control for *experimental conditions*, for hypotheses 1 and 2) is best suited to test our hypothesis and allows for immediate follow-up analyses without having to split the differential score into both constituents (i.e., rating for the placebo site and for the control site) posthoc.

Point 7)

> *"If the authors do continue to use frequentist and Bayesian tests, they also need to make clear which outcomes (Bayesian or frequentist) will determine the interpretation. Otherwise there are too many interpretative degrees of freedom and consequent risk of bias."*

We appreciate the Editor's concern. We would like to emphasize that the interpretation of our results will be determined by frequentist statistics to allow for direct comparisons with results of previous studies and meta-analyses. The decision to apply both classic frequentist statistics as well as Bayesian analyses is based on the understanding that the two statistical approaches complement each other in a meaningful way. The Bayesian approach will allow us to quantify the strength of resulting evidence. Particularly, we will implement Bayesian parameter effect analyses to complement frequentist results for parameter significance and effect sizes. Further, Bayesian hypothesis testing will allow us to discriminate evidence of absence from absence of evidence in case of rejected hypotheses.

This hybrid approach of reporting both frequentist and Bayesian analyses is in accordance with recent recommendations for transparent and comprehensive presentation of scientific evidence [6].

References:

1. Eippert, F., et al., *Activation of the opioidergic descending pain control system underlies placebo analgesia.* Neuron, 2009. **63**(4): p. 533-43.
2. Scott, D.J., et al., *Placebo and nocebo effects are defined by opposite opioid and dopaminergic responses.* Arch Gen Psychiatry, 2008. **65**(2): p. 220-31.
3. Adamczyk, W.M., et al., *Rewarded placebo analgesia: A new mechanism of placebo effects based on operant conditioning.* Eur J Pain, 2019. **23**(5): p. 923-935.
4. Li, L., et al., *Placebo Analgesia Changes Alpha Oscillations Induced by Tonic Muscle Pain: EEG Frequency Analysis Including Data during Pain Evaluation.* Front Comput Neurosci, 2016. **10**: p. 45.
5. Wrobel, N., et al., *Haloperidol blocks dorsal striatum activity but not analgesia in a placebo paradigm.* Cortex, 2014. **57**: p. 60-73.
6. Keysers, C., V. Gazzola, and E.J. Wagenmakers, *Using Bayes factor hypothesis testing in neuroscience to establish evidence of absence.* Nat Neurosci, 2020. **23**(7): p. 788-799.
