# Supplementary material for: Dopamine has no direct causal role in the formation of treatment expectations and placebo analgesia in humans
Source: PLoS Biol. 2024 Sep 24;22(9):e3002772. doi: 10.1371/journal.pbio.3002772 (PMC11421806; doi:10.1371/journal.pbio.3002772)
Supplement: Attachment — Submitted filename: ResponseLetter_final_220321.pdf [file pbio.3002772.s009.pdf]

Dear Dr. Pariente,

We would like to thank you and the reviewers for the positive evaluation of our manuscript entitled "The role of dopamine for positive treatment expectations and placebo analgesia" which we had submitted to PLOS Biology for consideration as a Preregistered Research Article.

A point-by-point reply to remaining comments of reviewer #3 is included below. All changes made to the manuscript and supplement are highlighted in yellow. We are confident that we could address all concerns and hope that the revised version will now be acceptable for publication in PLOS Biology.

Reviewers' comments:

Reviewer #1

This Stage 1 manuscript describes a comprehensive set of novel analytical approaches to examine the influence of dopaminergic mechanisms on the development of expectations, conditioning and placebo analgesia. It provides a novel approach not previously examined to determine pharmacological influences on the processes related to placebo analgesia

We would like to thank the reviewer for their positive evaluation of our manuscript and for highlighting the novelty and originality of our proposed study.

Reviewer #2:

The importance of the research question(s).
Very important as it can shed light on the interactions between expectations and pharmacological treatment in potentially all drug trials. The idea that dopamine may be a key transmitter for the formation of expectancies (rather than the inhibition of e.g., nociceptive signals) has not been tested in the rigorous, double-blind, systematic way proposed here.
The logic, rationale, and plausibility of the proposed hypotheses (does the manuscript provide a valid rationale for the proposed study, with clearly identified and justified research questions?)
Yes. The logic and rationale are clearly outlined. There is no reason to believe that the hypotheses are not valid, even if previous evidence is scarce and there is not much evidence to build on. Rather than a shortcoming I believe that the scarcity of previous data (there are some studies) points to the need for this type of study. The hypotheses are in line with previous data.
The soundness and feasibility of the methodology and analysis pipeline (including statistical power analysis where appropriate). Is the protocol technically sound and planned in a manner that will lead to a meaningful outcome and allow testing of the stated hypotheses?
The power had to be calculated on datasets that were not using the same design, and this may be a risk for the feasibility of getting results here. In fact, the effect size used to calculate power in this study was coming from a study with a different design (Scott et al. 2008), that was using raclopride PET ligands to measure DA binding in the brain as opposed to giving treatments with dopaminergic effects as suggested here. This means that the effect sizes used to calculate the power here are drawn from a very different context (that potentially has stronger effects). However, the authors have used a lower effect size in their power calculations to account for the potential lower effect sizes in this trial. This gives me reassurance that the analyses are likely to have enough power.
Whether the clarity and degree of methodological detail is sufficient to exactly replicate the proposed experimental procedures and analysis pipeline.
Yes. There is always risk that individual experimenters (and their behavior during application of cream) may affect the participant in a placebo analgesia experiment, especially since previous data suggest that non-verbal behavior will affect placebo responses in spite of clear instructions to experimenters (Kaptchuk et al. 2008). This is difficult to

control for and the authors have at least provided the verbal script for these interactions and clear instructions for the interactions.
Whether the authors have pre-specified sufficient outcome-neutral tests for ensuring that the results obtained are able to test the stated hypotheses, including positive controls and quality checks.
Yes.

We would like to thank the reviewer for their thorough evaluation of our manuscript and for highlighting the novelty, originality, methodological rigor and the broad implications of the results for both clinical routine and the conduction of RCTs.

Reviewer #3:

The authors propose a study designed to ask whether modulating dopamine systems through a pharmacological agonist (levodopa) or antagonist (sulpiride) modulates placebo analgesia by affecting treatment expectations. More specifically, they plan to administer levodopa, sulpiride, or vehicle prior to a placebo conditioning visit, in which participants will complete questionnaires, a pain calibration, and experience painful stimuli on a control site and a placebo site. Stimulus intensity will be lowered on the placebo site (VAS = 40, rather than VAS = 80), to induce expectations about pain reduction. The hypothesis is that the learning component that gives rise to expectations is dopamine-mediated. Although the hypotheses are not stated in directional terms, the figures indicate that the main hypothesis is that administration of levodopa will enhance expectations about pain relief, and thus that these participants will expect less pain and report larger reductions in pain (i.e. larger placebo effects) relative to a placebo group. In contrast, it is expected that a group that receives sulpiride will expect more pain than a placebo group, and have lower levels of placebo analgesia than either of the other groups. Placebo effects will be tested on days 2 and 8 following the initial visit. This study builds on previous work from this team, which indicated that administration of haloperidol reduced placebo-related activity in the striatum but not placebo analgesia itself. Thus they propose that rather than dopamine affecting pain processing itself, it is more related to learning, consistent with research outside the field of pain and placbeo. This is also consistent with findings of links between placebo-induced dopamine signalling in the nucleus accumbens being related to striatal reward responses, dopamine-related personality traits, and gray matter density in the striatum.
In principle, I think that continued investigation of the role of dopamine in placebo and pain modulation is important for the field.

We would like to thank the reviewer for acknowledging the importance of the proposed study. We appreciate their constructive comments and the opportunity to clarify the scope of our study as well as the choice and details of the experimental protocol. We address each of the comments below.

However, given that this is a registered report, I do not think that this study design will allow for incontrovertible evidence as to the precise contribution of dopamine to placebo, even if the results are in the hypothesized directions. First, although the authors previously found no impact of dopamine modulation on placebo analgesia, that does not mean that the administration cannot affect pain. Thus during the conditioning visit, dopamine administration might affect the calibration procedure. If the calibration procedure is completed prior to drug administration (this is not clear from the design), then it still could affect the extent to which an individual perceives differences in pain between the placebo site and the control site. This wouldn't be due to learning, but due to differences in responses to noxious stimuli. The only way to control for this would be to measure pain sensitivity before and after drug administration. If there is no difference in pain sensitivity but an effect on learning, then this can be attributed to dopamine modulating learning.

We agree that the potential influence of dopamine on pain sensitivity more generally is indeed an important point. However, we would like to stress that our methodological approach accounts and controls for such an effect in several ways.

First, although results regarding the role of dopamine in affective pain processing and pain chronification have been inconsistent, previous experimental investigations showed no effect of the augmentation or blockade of dopaminergic signaling on heat pain perception. In our own work (cited on page 3 in the manuscript), we found that 2 mg of the potent dopamine antagonist haloperidol does not influence thermal pain ratings when compared to a saline control (Wrobel et al., 2014). Others did also not find an effect of pharmacologic modulation of dopaminergic signaling on thermal pain sensitivity with drugs enhancing dopaminergic signaling or leading to dopamine depletion or blockade in healthy volunteers (Becker et al., 2013) and in patients with neuropathic pain (Skyt et al., 2018). On the backdrop of these previous observations, a general effect on pain sensitivity seems unlikely. Second, a general effect of dopaminergic modulation on pain sensitivity would be reflected in a main effect of *medication*. Yet our main hypothesis focuses on the interaction effect of *medication* (L-dopa, sulpiride, inactive pill) and *experimental condition* (placebo vs. control). Third, given that the *experimental condition* (placebo vs. control) is varied in a within-subject design, a general effect on pain sensitivity would affect both experimental conditions and not bias placebo analgesia as our main outcome that is defined as the difference in pain ratings on the control as compared to the placebo treated skin. Finally, we would like to clarify that the calibration procedure will be performed shortly before the conditioning session when both drugs are already expected to rise to their peak and effective plasma concentration. This will be after the second pill intake and before cream application. We have now added the exact time point of the calibration procedure into the method section of the manuscript. The calibration procedure should therefore take into account any potential changes in pain sensitivity induced by the drug. As outlined in the method section (e.g., Experimental Procedure), the temperature levels needed to achieve the intended pain perceptions (corresponding to VAS 40 and VAS 80) will reflect such an effect and could – if necessary – be included as a covariate in the analyses.

Although we believe that these measures are sufficient to account and control for an acute effect of the pharmacological modulations of thermal pain sensitivity, we will – in response to the reviewer's suggestion - assess thermal pain thresholds (i.e., method of limits, Fruhstorfer et al., 1976) prior to the pharmacological modulation and immediately before the calibration session to allow for a direct assessment of drug effects on pain sensitivity.

Given that other studies suggest links between placebo and other processes that are more commonly linked to dopamine, why don't the authors include a learning or reward task on day 1 alongside conditioning? This would provide a positive control that the pharmacological manipulation is effective.

We would like to clarify that the main scope of this investigation is to investigate the general contribution of dopaminergic signaling to the generation of positive treatment expectations and resulting placebo analgesia. Dopaminergic transmission has been implicated in a number of processes including learning and reward processing but also affective valuation and decision-making which could in principle underlie any effect we find. However, we think that at this stage our design should be optimized to answer our primary question which could be followed up by studies that dissect the relative contribution of dopamine-related processes. We have therefore chosen drug dosages that have proven to effectively alter dopamine-related behavior by an increase dopaminergic tone after administration of 100 mg of L-Dopa (Delaveau et al., 2005; Pessiglione et al., 2006; Weis et al., 2012) or to decrease dopaminergic signaling with 400 mg Sulpiride (Dodds et al., 2009; Eisenegger et al., 2014; Ojala et al., 2018; Weber et al., 2016) in the past.

We agree that a validation of successful pharmacological modulation is important. We chose to validate effective modulation of dopaminergic tone by assessing common physiological measures of modulation. To monitor an effective blockade of D2-Receptors by sulpiride, we exploit the D2-

receptor dependent release of the protein prolactin in the pituitary (Ben-Jonathan & Hnasko 2001). Inhibition of D2 receptors in the pituitary leads to a quick surge in prolactin serum levels. This has been shown for a single-dose intake of sulpiride as well as other antipsychotic drugs that target the D2-receptor. Thus, we use prolactin as a surrogate marker for successful D2 manipulation in the brain. For the L-Dopa-group, we will determine L-Dopa in the blood, as we and others did before to check effective serum levels for pharmacologic manipulation. (Weis et al., 2012; Zunhammer, Gerardi & Bingel, 2018).

We would also like to point out that adding another task to explore the influence of reward or learning after the conditioning session would come at different costs. First, we would risk drug levels to fall below the minimum effective concentration again before participants complete the experiment, particularly for the L-Dopa manipulation, since the drug's half-life is only ~1,5 hours (Hsu et al., 2015) and behavioral effects are expected to be highest around the serum level peak, when we are performing conditioning. Second, combining the conditioning session which already comprises the learning of analgesic rewards with an additional reward-learning task would lead to complex carry-over and interaction effects between the task which would jeopardize the main aim and interpretation of our study. Based on these considerations we decided to focus on the proof-of-concept aspect of this study and to ensure the validity check for the successful pharmacological modulations based on the prolactin measurements.

I also think that the decision to test the magnitude of placebo on day 2 and 8 is not well justified in the paper. If the drug modulates learning and expectations, why can you not test placebo effects on the first visit day? It is not clear to me why these should necessarily be long-lasting effects; there is no specific hypothesis about memory or consolidation offered in the paper.

We would like to clarify that our main hypothesis (*Hypothesis 1*) is centered on the question how the dopaminergic modulation during the conditioning session influences the generation of positive treatment expectations and resulting placebo analgesia. If placebo analgesia would be tested on day one, potential differences in placebo analgesia between groups could be driven by processes in the conditioning session and/or the actual test session. Although previous research renders the latter effect unlikely, (Becker et al., 2013; Skyt et al., 2018; Wrobel et al., 2014), we want to exclude such effects by separating the influence of the pharmacological modulation from the placebo analgesia test session. This seems especially relevant to avoid such effects in the sulpiride group, as the half-life is over 8 hours. There is sufficient evidence from previous studies indicating that conditioning on the previous day results in significant placebo analgesic effects in test sessions performed on the next day (Amanzio & Benedetti, 1999; Benedetti et al., 1999; Kessner et al.; 2014; Zunhammer et al., 2017).

We would like to stress that the assessment of treatment expectations and placebo analgesia on day 8 is purely explorative and motivated by the substantial clinical implications of potential changes in these effects over time. Given the explorative nature of this additional investigation we would like to refrain from formulating specific hypotheses for day 8.

Finally, there are highlighted sections in the main manuscript that are redundant, and three figures are missing captions. These are also highlighted, which suggests the authors may have turned in an incomplete draft accidentally.

We apologize if the submission was indeed incomplete. Following the suggestions of the Academic Editor Figures 2-4 were inserted subsequently and therefore highlighted in the manuscript. We

have now inserted the figure legends corresponding to the formatting guidelines of PLOS Biology in the manuscript text immediately after the first paragraph in which the figure is cited.

References

Amanzio, M., & Benedetti, F. (1999). Neuropharmacological dissection of placebo analgesia: expectation-activated opioid systems versus conditioning-activated specific subsystems. *Journal of Neuroscience*, *19*(1), 484-494.

Becker, S., M. Ceko, M. Louis-Foster, N. M. Elfassy, M. Leyton, Y. Shir, and P. Schweinhardt. 2013. 'Dopamine and pain sensitivity: neither sulpiride nor acute phenylalanine and tyrosine depletion have effects on thermal pain sensations in healthy volunteers', *PLoS One*, 8: e80766.

Benedetti, F., Amanzio, M., Baldi, S., Casadio, C., & Maggi, G. (1999). Inducing placebo respiratory depressant responses in humans via opioid receptors. *European Journal of Neuroscience*, *11*(2), 625-631.

Ben-Jonathan, N., and R. Hnasko. 2001. 'Dopamine as a prolactin (PRL) inhibitor', *Endocr Rev*, 22: 724-63.

Delaveau, P., P. Salgado-Pineda, B. Wicker, J. Micallef-Roll, and O. Blin. 2005. 'Effect of levodopa on healthy volunteers' facial emotion perception: an FMRI study', *Clin Neuropharmacol*, 28: 255-61.

Dodds, C. M., L. Clark, A. Dove, R. Regenthal, F. Baumann, E. Bullmore, T. W. Robbins, and U. Muller. (2009). 'The dopamine D2 receptor antagonist sulpiride modulates striatal BOLD signal during the manipulation of information in working memory', *Psychopharmacology (Berl)*, 207: 35-45.

Eisenegger, C., M. Naef, A. Linssen, L. Clark, P. K. Gandamaneni, U. Muller, and T. W. Robbins. 2014. 'Role of dopamine D2 receptors in human reinforcement learning', *Neuropsychopharmacology*, 39: 2366-75.

Fruhstorfer H, Lindblom U, Schmidt W. Method for quantitative estimation of thermal thresholds in patients. *Journal of Neurology*, Neurosurgery & Psychiatry. 1976;39(11):1071-5.

Hood, S. D., J. P. Potokar, S. J. Davies, D. A. Hince, K. Morris, K. M. Seddon, D. J. Nutt, and S. V. Argyropoulos. 2010. 'Dopaminergic challenges in social anxiety disorder: evidence for dopamine D3 desensitisation following successful treatment with serotonergic antidepressants', *J Psychopharmacol*, 24: 709-16.

Hsu, A., H. M. Yao, S. Gupta, and N. B. Modi. 2015. 'Comparison of the pharmacokinetics of an oral extended-release capsule formulation of carbidopa-levodopa with immediate-release carbidopa-levodopa, sustained-release carbidopa-levodopa, and carbidopa-levodopa-entacapone (*Journal of Clinical Pharmacology*, 55: 995-1003.

Kessner, S., Forkmann, K., Ritter, C., Wiech, K., Ploner, M., & Bingel, U. (2014). The effect of treatment history on therapeutic outcome: psychological and neurobiological underpinnings. *PloS One*, *9*(10).

Mehta, M. A., A. J. Montgomery, Y. Kitamura, and P. M. Grasby. 2008. 'Dopamine D2 receptor occupancy levels of acute sulpiride challenges that produce working memory and learning impairments in healthy volunteers', *Psychopharmacology (Berl)*, 196: 157-65.

Ojala, K. E., L. K. Janssen, M. M. Hashemi, M. H. M. Timmer, D. E. M. Geurts, N. P. Ter Huurne, R. Cools, and G. Sescousse. 2018. 'Dopaminergic Drug Effects on Probability Weighting during Risky Decision Making', *eNeuro*, 5.

Pessiglione, M., B. Seymour, G. Flandin, R. J. Dolan, and C. D. Frith. 2006. 'Dopamine-dependent prediction errors underpin reward-seeking behaviour in humans', *Nature*, 442: 1042-5.

Schlegel, S., R. Schlosser, C. Hiemke, O. Nickel, A. Bockisch, and K. Hahn. 1996. 'Prolactin plasma levels and D2-dopamine receptor occupancy measured with IBZM-SPECT', *Psychopharmacology (Berl)*, 124: 285-7.

Sekine, M., J. Maeda, H. Shimada, T. Nogami, R. Arakawa, H. Takano, M. Higuchi, H. Ito, Y. Okubo, and T. Suhara. 2011. 'Central nervous system drug evaluation using positron emission tomography', *Clin Psychopharmacol Neurosci*, 9: 9-16.

Skyt, I., K. Moslemi, C. Baastrup, K. Grosen, F. Benedetti, G. L. Petersen, D. D. Price, K. T. Hall, T. J. Kaptchuk, P. Svensson, T. S. Jensen, and L. Vase. 2018. 'Dopaminergic tone does not influence pain levels during placebo interventions in patients with chronic neuropathic pain', *Pain*, 159: 261-72.

Weber, S. C., B. Beck-Schimmer, M. E. Kajdi, D. Muller, P. N. Tobler, and B. B. Quednow. 2016. 'Dopamine D2/3- and mu-opioid receptor antagonists reduce cue-induced responding and reward impulsivity in humans', *Transl Psychiatry*, 6: e850.

Weis, T., S. Puschmann, A. Brechmann, and C. M. Thiel. 2012. 'Effects of L-dopa during auditory instrumental learning in humans', *PLoS One*, 7: e52504.

Wrobel, N., K. Wiech, K. Forkmann, C. Ritter, and U. Bingel. 2014. 'Haloperidol blocks dorsal striatum activity but not analgesia in a placebo paradigm', *Cortex*, 57: 60-73.

Zunhammer, M., Ploner, M., Engelbrecht, C., Bock, J., Kessner, S. S., & Bingel, U. (2017). The effects of treatment failure generalize across different routes of drug administration. *Science Translational Medicine*, *9*(393).

Zunhammer, M., M. Gerardi, and U. Bingel. 2018. 'The effect of dopamine on conditioned placebo analgesia in healthy individuals: a double-blind randomized trial', *Psychopharmacology (Berl)*, 235: 2587-95.
