# Supplementary material for: Dopamine has no direct causal role in the formation of treatment expectations and placebo analgesia in humans
Source: PLoS Biol. 2024 Sep 24;22(9):e3002772. doi: 10.1371/journal.pbio.3002772 (PMC11421806; doi:10.1371/journal.pbio.3002772)
Supplement: Attachment — Submitted filename: Responses to Reviewer Comments_submitted.docx [file pbio.3002772.s010.docx]

Responses to the reviewer comments on the Stage 2 manuscript

Responses from the team of authors are positioned below each comment (italic font).

Reviewer #2: The article is a clear report of the results from the pre-registered study on the role of dopamine in placebo analgesia. The authors have made a throrough analysis of the data and, perhaps due to the negative finding, performed many sub-analyses to explore the data extensively. The conclusions are in line with the results and are not over-reaching. I find the manuscript suitable for publication. I would like to congratulate the authors to this large undertaking, and well-balanced discussion of the results.

*We would like to thank the reviewer for assessing our manuscript and ensuring that all results and findings, including the exploratory observations, are presented adequately. We thank you for your time and effort.*

Reviewer #3: I am really glad the authors conducted this study, as the field has speculated for some time about the role of dopamine in placebo effects due to a single paper and no comprehensive tests of the hypotheses. Doing this through a registered report really provides definitive evidence of there being no substantial relationship between placebo analgesia and dopamine, building on earlier findings from the team (Wrobel et al.). The authors followed their preregistered analyses and study designs, and conducted appropriate additional exploratory analyses that are important for any readers who may still believe that there is a hidden impact of dopamine.

My only minor comment - and it is truly minor - is that there are post-hoc interpretations in the section under "Considering absolute pain intensity" that imply a medication x mean pain interaction, despite the fact that the interaction was not significant (lines 625-630, figure 7E). Given that there is no interaction with medication, it seems unnecessary to explore the slopes for the different medication groups. I recognize that the frequentist p-value is .06, so perhaps they felt a need to explore the potential interaction, but Bayesian models could also be used to see whether the interaction is meaningful.

Thank you again for the opportunity to review this paper, and for your contribution to the field.

-Lauren Atlas

*We are grateful and highly appreciative of the reviewer’s time and effort in thoroughly evaluating our manuscript. We agree that depicting statistical details about the slopes for different medication groups is not necessarily warranted, given the insignificant medication x mean pain interaction. We followed the reviewer’s considerations by removing the referenced sentence in the manuscript entirely (lines 625-630).*
